# TSP-1 promotes glomerular mesangial cell proliferation and extracellular matrix secretion in Thy-1 nephritis rats^☆^

**DOI:** 10.1016/S1674-8301(11)60053-5

**Published:** 2011-11

**Authors:** Wen Qiu, Yan Li, Jianbo Zhou, Chenhui Zhao, Jing Zhang, Kai Shan, Dan Zhao, Yingwei Wang

**Affiliations:** a Department of Microbiology and Immunology, Nanjing Medical University, Nanjing, Jiangsu 210029, China;; b School of Clinical Medicine, Jiangsu University, Zhenjiang, Jiangsu 212013, China.

**Keywords:** Thy-1 nephritis, glomerular mesangial cells (GMCs), proliferation, extracellular matrix, thrombospondin-1 (*TSP-1*)

## Abstract

The proliferation of glomerular mesangial cells (GMC) and secretion of the extracellular matrix (ECM) in rat with Thy-1 nephritis (Thy-1N) resembling human mesangioproliferative glomerulonephritis have been explored for many years; however, the molecular mechanisms of GMC proliferation and ECM production remain unclear. Our previous studies have demonstrated that the thrombospondin-1 (*TSP-1*) gene was involved in mediating rat GMC proliferation and ECM synthesis induced by sublytic C5b-9 *in vitro*. In the present study, the roles of the *TSP-1* gene in GMC proliferation, ECM production, and urinary protein secretion in Thy-1N rats were determined by using *TSP-1* small hairpin RNA, and the results revealed that silencing of the *TSP-1* gene in rat renal tissues could diminish GMC proliferation (*P* < 0.01) and ECM secretion (*P* < 0.01) as well as urinary protein secretion (*P* < 0.05) in Thy-1N rats. Together, the current findings suggested that *TSP-1* gene expression was required for GMC proliferation and ECM production in Thy-1N rats.

## INTRODUCTION

Mesangioproliferative glomerulonephritis is a disease with a high incidence in humans[1]. Considerable evidence indicates that the proliferation of glomerular mesangial cells (GMC) in mesangioproliferative glomerulonephritis appears to be crucial in the secondary production of the mesangial extracellular matrix (ECM)[2],[3]. Although the deposits of complement C5b-9 complexes have been shown in the glomeruli of patients with mesangioproliferative glomerulonephritis[4],[5], the role of C5b-9 has not been fully understood until now. Rat Thy-1 nephritis is an animal model for human mesangioproliferative glomerulonephritis[6]–[8]. Thy-1 antibody can bind to Thy-1 antigen present on the GMC membrane, forming immune complexes, and then activate the complement system, causing GMC pathological lesions[9],[10]. Several reports have demonstrated that renal pathological lesions in Thy-1 nephritis are C5b-9-dependent and neutrophil-independent[6],[11],[12].

It is well known that C5b-9 injury to nucleated cells is almost sublytic because cells can eliminate membrane-inserted C5b-9 through endocytosis and/or shedding by homologous restriction factors. Sublytic C5b-9 attack can result in diverse events including cellular apoptosis, necrosis, proliferation and cytokine releases[13]–[16]. Early data from our experiments have confirmed that GMC proliferation and ECM production were markedly increased in GMC stimulated by sublytic C5b-9 *in vitro*[17],[18], indicating that sublytic C5b-9, as a trigger, could play an important role in proliferative pathological changes in Thy-1 nephritis.

Thrombospondin-1 (TSP-1), as a multifunctional protein, plays essential roles in regulating cell proliferation and cell-ECM interaction[19]–[21]. During human and animal glomerulonephritis, TSP-1 is transiently highly expressed at sites of injury by various endogenous renal and inflammatory cell types[21]–[25]. Mounting evidence from previous studies suggests that TSP-1 can bind to transforming growth factor-β1 (TGF-β1) latent complexes, and then activate TGF-β1, which promotes the synthesis of matrix proteins[26]. Our published reports manifested that TSP-1 expression and TGF-β1 activation as well as GMC proliferation and ECM accumulation were increased in GMCs induced by sublytic C5b-9. Treatment of GMC with the GGWSHW peptide (a selective inhibitor of TSP-1) could block the interaction between TSP-1 and TGF-β1 and subsequent TGF-β1 activation as well as ECM production[17],[18], and treatment of GMCs with TSP-1 small hairpin RNA (shRNA) could suppress GMC proliferation (data not shown), implying that TSP-1 could play a role in GMC proliferation and ECM secretion in GMCs exposed to sublytic C5b-9. However, the role of the *TSP-1* gene in mediating GMC proliferation and ECM synthesis in renal tissues of Thy-1 nephritic rats has not been fully understood, although previous study showed that transfection with oligonucleotide against *TSP-1* could inhibit glomerular ECM accumulation[27].

In the present study, the plasmids of *TSP-1* shRNA (shTSP-1) were constructed and transfected into rat kidneys via renal artery perfusion followed by electroporation, and then anti-Thy-1 antibody was injected into the rats to induce Thy-1 nephritis. Corresponding experiments were then performed to determine the effects of *TSP-1* gene knockdown on GMC proliferation and ECM production as well as urinary protein excretion in rats with Thy-1 nephritis.

## MATERIALS AND METHODS

### Reagents and animals

Polyclonal antibodies against cyclin D2, collagen IV and TSP-1, and monoclonal antibody against Thy-1 antigen (OX-7) and fibronectin were purchased from Santa Cruz Biotechnology (Santa Cruz, CA, USA). Monoclonal antibody against proliferative cell nuclear antigen (PCNA) was supplied by Thermo (Fremont, CA, USA). Monoclonal antibody against β-actin, HRP-conjugated anti-mouse IgG antibody, 20×LumiGLO reagent® and 20×peroxide were purchased from Cell Signaling Technology (Danvers, USA). HRP-conjugated anti-goat IgG antibody and FITC-linked anti-mouse IgG antibody were from Dako (Glostrup, Denmark) and KPL (Gaithersburg, MD, USA), respectively. 5-bromo-2-deoxyuridine (BrdU) was purchased from Sigma (St. Louis, MO, USA), and antibody against BrdU was from Thermo. The shRNA expression plasmids of pGCsi.U6.neo.RFP were from Genechem (Shanghai, China).

Rabbit polyclonal antibody against Thy-1 antigen was prepared according to previous documents[28],[29]. Male Sprague-Dawley (SD) rats were from B&K Universal Ltd. (Shanghai, China). The animal study protocol was approved by the local institutional review board and carried out in accordance with the Guide for the Care and Use of Laboratory Animals published by the U.S. National Institutes for Health (NIH Publication No. 85-23, Revised 1996).

### Construction of shRNA expression plasmids

To silence rat *TSP-1* gene, we designed different shRNA sequences against *TSP-1* (NM_001013062) mRNA. The expression plasmids of shTSP-1 were constructed by using pGCsi.U6.neo.RFP (*Xho*I and *Eco*RI). The most effective shRNA expression plasmids (CTCAATGAACGAGACAACT) against the *TSP-1* gene were chosen for further experiments. Meanwhile, the control shRNA (shCTR) expression plasmids (CTTGCATTGCATCCTCATT) were produced as a negative control.

### Establishment of Thy-1 nephritis model in rats and the experimental design

**Fig. 1:**
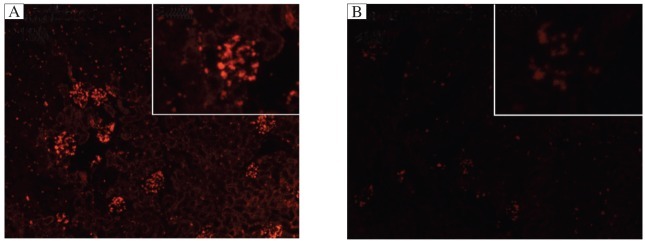
The transfection efficiency of shRNA expression plasmids *in vivo* (Magnification: ×100; inset: ×400). The plasmids of shCTR were transfected into rat kidneys *via* renal artery perfusion followed by electroporation. Frozen sections were used to observe the expression of red fluorescent protein (RFP) in the glomeruli of rats at 72 h after transfection under a fluorescence microscope to evaluate transfection efficiency. A: transfected samples. B: non-transfected sampes. The results showed that shRNA expression plasmids could be effectively delivered to the glomeruli of rats by renal artery perfusion followed by electroporation.

To confirm the roles of TSP-1 in mediating GMC proliferation and ECM secretion in Thy-1 nephritis, we randomly divided male SD rats (180-200 g) into 4 groups (*n* = 6 in each group), namely: 1) the Thy-1 nephritis group in which animals were given anti-Thy-1 antibody (0.75 mL/100 g body weight) by a single intraperitoneal injection; 2) the shTSP-1 + Thy-1 nephritis group; 3) the shCTR + Thy-1 nephritis group, and 4) the NS group in which the rats were injected intraperitoneally with normal rabbit serum (0.75 mL/100 g body weight). The rats allocated to group 2 and 3 were treated with modification as described previously[6]. The cortexes of rats were collected on d 7 after nephritis induction upon sacrifice. Red fluorescence protein (RFP) expression was observed to define the efficiency of transferring the plasmids into the kidneys (***Fig. 1***), and the efficiency of silencing *TSP-1* gene expression was evaluated by immunoblotting assays. Part of renal tissues was also examined using immunoblotting analysis for the expression of cyclin D2, PCNA, fibronectin and collagen IV. In addition, the proliferative changes of GMC were determined by light and electron microscopy as well as by immunohistochemisty and immunofluorescence staining.

### Immunoblotting assays

Rat renal tissues were lysed using the RIPA lysis buffer. Equal amounts (20 µg/lane) of protein were subjected to SDS-PAGE. Immunoblotting analysis was performed as described previously[6]. β-actin was used as an internal control of protein loading and the level of protein in each group was expressed relative to the control group.

### BrdU incorporation analysis

To observe GMC proliferation of the rats with Thy-1 nephritis, we assessed glomerular cells by identification of cellular incorporation of BrdU into nuclear DNA. Beginning at 24 h prior to injection of anti-Thy-1 antibody, SD rats received an intraperitoneal injection of BrdU at a dose of 5 mg/100 g body weight every 12 h throughout disease progression to label proliferative cells[30]–[32]. BrdU uptake into the glomeruli was detected by immunofluorescence techniques as described below.

### Immunohistochemical staining

Paraffin-embedded rat renal tissues were examined by immunohistochemical staining to determine the expression of TSP-1, PCNA and collagen IV and Thy-1 antigen (OX-7, as a marker of GMC). Firstly, sections (4 µm) were deparaffinized and incubated with purified antibodies. Then, the sections were incubated with HRP-conjugated secondary antibody, and were visualized *via* chromogen 3′-diaminobenzidine (DAB) staining. The number of glomerular PCNA- or OX-7-positive cells was counted in a double-blinded manner by observing 100 glomerular cross-sections from each rat, and was expressed as the mean number per glomerular cross-section as described previously[6].

### Immunofluorescence staining

The frozen sections (4 µm) of rat renal tissues were stained with antibodies to BrdU, and then incubated with the corresponding fluorescein isothiocyanate (FITC)-conjugated secondary antibody. The number of glomerular BrdU-positive cells was detected according to the above-mentioned method. The number of glomerular BrdU-positive cells was expressed as the mean number per glomerular cross-section.

### Proliferative change examination

For light microscopy, the histological sections (4 µm) of all renal cortex samples on d 7 after nephritis induction were stained with H&E, and 100 glomerular cross-sections from each rat were examined according to the same method[6]. For electron microscopy, ultrathin sections of all samples on d 7 were stained with uranyl acetate and lead citrate, and the ultrastructural changes were observed[28],[33].

### Urinary protein excretion

The urine samples of rats in different groups were collected on d 7 after the induction of nephritis. The contents of urinary protein (mg/24 h) of rats were measured by the total protein UC FS (DiaSys Diagnostic Systems, Holzheim, Germany).

### Statistical analysis

Data are presented as mean±SD. One-way ANOVA was used to determine significant differences among groups. Where significant differences were found, individual comparisons were made between groups using the Student's *t* test and adjusting the critical value according to the Bonferroni method. *P* < 0.05 was considered significant.

## RESULTS

### The effect of shTSP-1 on TSP-1 gene expression in the renal tissues of rats with Thy-1 nephritis

**Fig. 2:**
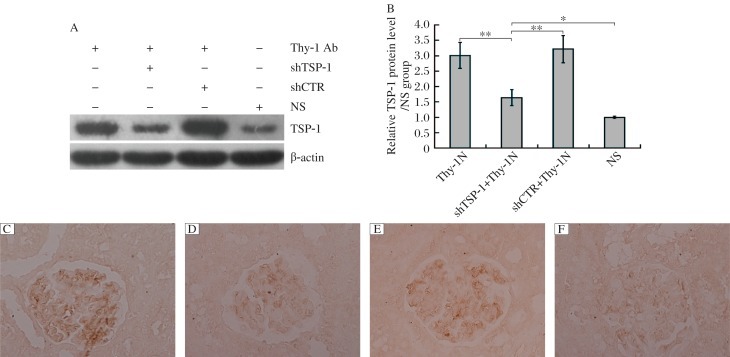
The effects of shTSP-1 on renal TSP-1 expression in Thy-1 nephritis rats. The rats were divided into following 4 groups: the Thy-1 nephritis group, the shTSP-1 + Thy-1 nephritis group, the shCTR + Thy-1 nephritis group, and the NS group as described in “MATERIALS AND METHODS”. The renal expression of TSP-1 protein in different groups of rats was detected on d 7 after Thy-1 nephritis induction. Immunoblot analysis showed that shTSP-1, but not shCTR, could reduce the expression of TSP-1 in the renal tissues of Thy-1 nephritis rats. A: immunblotting results TSP-1 expression. B: statistical anysis of imunoblotting results. Data are mean±SD (*n* = 6 in each group). *P < 0.05,**P < 0.01. Immunohistochemical staining (Paraffin-embedded sections, Original magnification: ×400) exhibited that shTSP-1 could reduce the expression of TSP-1 in the glomeruli of Thy-1 nephritis rats compared with shCTR treatment. C: the Thy-1 nephritis group. D: the shTSP-1 + Thy-1 nephritis group. E: the shCTR + Thy-1N nephritis group. F: the NS group.

In order to explore the roles of *TSP-1* gene activation in the pathological changes of Thy-1 nephritis in rats, we transfected the plasmids of shTSP-1 and shCTR respectively, into rat kidneys followed by Thy-1 nephritis induction. The rats were divided into following 4 groups: 1) the Thy-1 nephritis group; 2) the shTSP-1 + Thy-1 nephritis group; 3) the shCTR + Thy-1 nephritis group; 4) the NS group. The renal expression of TSP-1 protein in different groups of rats was examined on d 7 after Thy-1 nephritis induction, and the results showed that the expression of the *TSP-1* gene was obviously increased in the renal tissues of Thy-1 nephritis rats, and that shTSP-1 could effectively reduce the expression of TSP-1 in the renal tissues of Thy-1 nephritis rats, while shCTR had no significant effect on renal TSP-1 expression (***Fig. 2***).

### The roles of renal TSP-1 expression in GMC proliferation in rats with Thy-1 nephritis

**Fig. 3:**
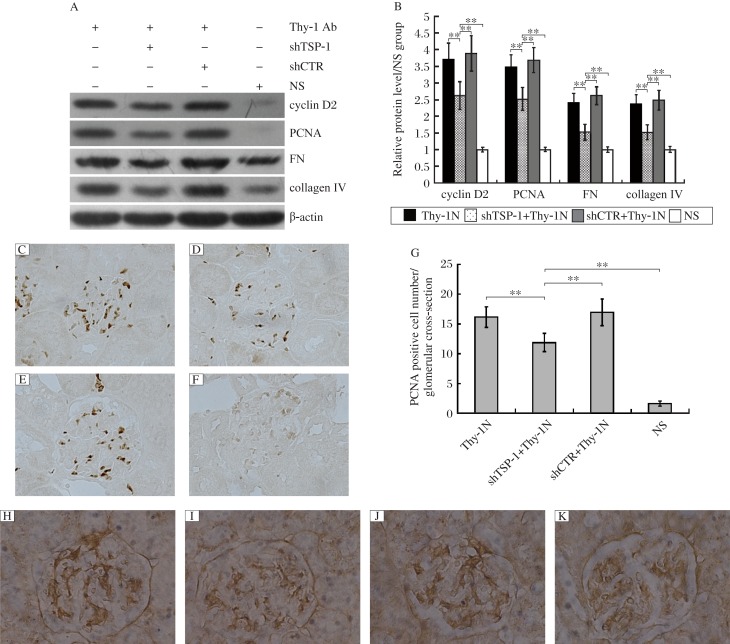
The effects of *TSP-1* gene silencing on relative gene expression in Thy-1 nephritis rats. Immunoblotting analysis showed that shTSP-1 reduced the levels of cyclin D2, proliferative cell nuclear antigen (PCNA), fibronectin (FN) and collagen IV in the renal tissues of Thy-1 nephritis rats on d 7 after nephritis induction, while shCTR had no significant effect on the expression of cyclin D2, PCNA, FN and collagen IV. A: immuoblotting results of cyclin D2, PCNA, FN and collagen expression. B: statistieal analysis of immunoblotting results. Immunohistochemistry staining (IH) for PCNA was performed (Paraffin-embedded sections, Original magnification: ×400) and analysis showed that the number of PCNA-positive cells in the shTSP-1 + Thy-1 nephritis group on d 7 was lower than that in the shCTR + Thy-1 nephritis and the Thy-1 nephritis groups. C: the Thy-1N nephritis group. D: the shTSP-1 + Thy-1 nephritis group. E: the shCTR + Thy-1N nephritis group. F: the NS group. G: the statistieal analysis of PCNA detected by IH staining. Immunohistochemistry staining (Paraffin-embedded sections, Original magnification: ×400) showed that the expression of collagen IV in the glomeruli of rats in the shTSP-1 + Thy-1 nephritis group was lower than that in the shCTR + Thy-1 nephritis group and the Thy-1 nephritis group on d 7. H: the Thy-1 nephritis group. I: the shTSP-1 + Thy-1 nephritis group. J: the shCTR + Thy-1N nephritis group. K: the NS group. Data are represented as mean±SD (*n* = 6 per group). **P < 0.01.

**Fig. 4:**
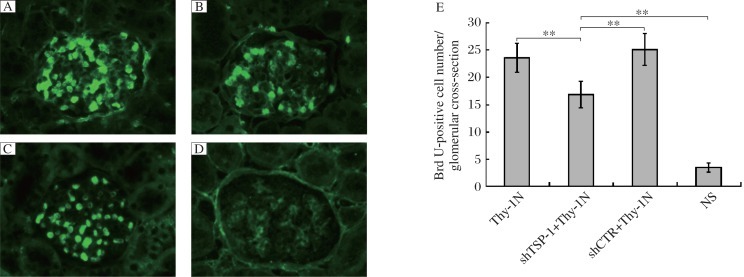
The effects of *TSP-1* gene knockdown on the number of glomerular BrdU-positive cells inThy-1 nephritis rats (Original magnification: ×400). SD rats were divided into the Thy-1 nephritis, shTSP-1 + Thy-1 nephritis, shCTR + Thy-1 nephritis and NS groups as described in “MATERIALS AND METHODS”. The number of glomerular BrdU-positive cells in the shTSP-1 + Thy-1 nephritis group was lower than that in the shCTR + Thy-1 nephritis and Thy-1 nephritis groups on d 7 after nephritis induction. Immunofluorescence (IF) staining results for BrdU (Frozen sections) were shown. A: the Thy-1 nephritis group. B: the shTSP-1 + Thy-1 nephritis group. C: the shCTR + Thy-1N nephritis group. D: the NS group. E: statistical analysis of IF. Data are represented as mean±SD (*n* = 6 per group). **P < 0.01.

**Fig. 5:**
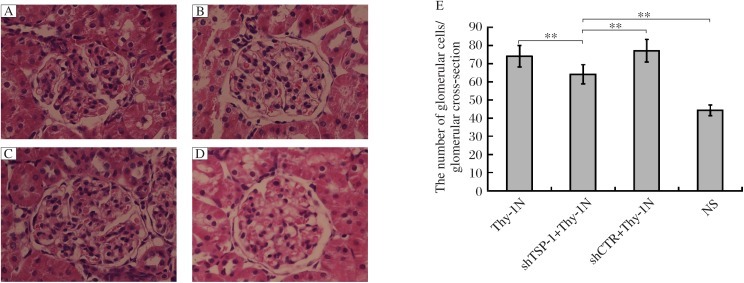
The effects of shTSP-1 on the number of glomerular cells in Thy-1 nephritis rats (Original magnification: ×400). SD rats were divided into Thy-1 nephritis, shTSP-1 + Thy-1 nephritis, shCTR + Thy-1 nephritis and NS groups as described in “MATERIALS AND METHODS”. The number of glomerular cells was reduced in shTSP-1 + Thy-1 nephritis group on d 7 after Thy-1 nephritis establishment, compared with shCTR + Thy-1 nephritis and Thy-1 nephritis groups detected by H&E staining (Paraffin-embedded sections). A: the Thy-1 nephritis group. B: the shTSP-1 + Thy-1 nephritis group. C: the shCTR + Thy-1N nephritis group. D: the NS group. E: statistical analysis of HE staining. Data were represented as means ± SD (*n* = 6 per group). **P < 0.01.

**Fig. 6:**
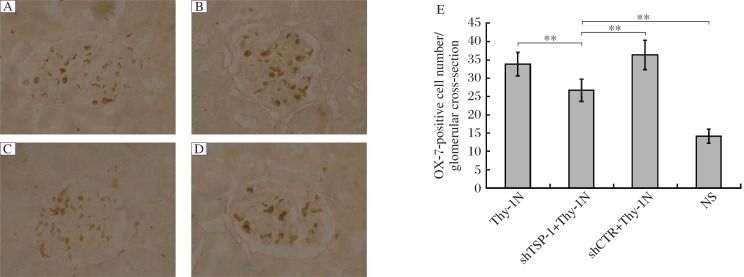
The effects of silencing the *TSP-1* gene on GMC number in Thy-1 nephritis rats (Original magnification: ×400). SD rats were divided into the Thy-1 nephritis, shTSP-1 + Thy-1 nephritis, shCTR + Thy-1 nephritis and NS groups. showed The number of OX-7-positive cells (i.e. GMC) in the shTSP-1 + Thy-1 nephritis group was also less than that in shCTR + Thy-1 nephritis and Thy-1 nephritis groups on d 7. Immunohistochemistry staining (IH) results for Thy-1 antigen (OX-7) (Paraffin-embedded sections) are shown. A: the Thy-1 nephritis group. B: the shTSP-1 + Thy-1 nephritis group. C: the shCTR + Thy-1N nephritis group. D: the NS group. E: the statistical analysis of OX-7 detected by IH. Data are represented as mean±SD (*n* = 6 per group). **P < 0.01.

**Fig. 7:**
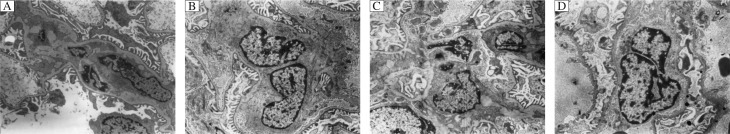
The effects of shTSP-1 on GMC proliferation and ECM production in Thy-1 nephritis rats (Original magnification: ×5,000). Electron microscopy showed that the rats in the shTSP-1 + Thy-1 nephritis group on d 7 after nephritis induction exhibited less GMC proliferation and ECM secretion compared with the shCTR + Thy-1 nephritis group and Thy-1 nephritis group. A: the Thy-1 nephritis group. B: the shTSP-1 + Thy-1 nephritis group. C: the shCTR + Thy-1N nephritis group. D: the NS group.

To determine the roles of the *TSP-1* gene in GMC proliferation of Thy-1 nephritis rats, we examined the levels of cyclin D2 and PCNA protein in the renal tissues of the above-mentioned rats on d 7, and the results manifested that shTSP-1, but not shCTR, could suppress the expression levels of cyclin D2 and PCNA (***Fig. 3***). Meanwhile, the number of proliferating glomerular cells and the total number of glomerular cells in the shTSP-1 + Thy-1 nephritis group were obviously lower than those in the shCTR + Thy-1 nephritis and Thy-1 nephritis groups (***Fig. 4*** and ***Fig. 5***). It is worth mentioning that the changes of glomerular cell numbers were indeed due to the changes of GMC number because staining with anti-Thy-1 antibody (OX-7) was used to label GMC, and similar results were obtained (***Fig. 6***). On the other hand, the glomeruli of rats in the shTSP-1 + Thy-1 nephritis group exhibited less GMC proliferation relative to the shCTR + Thy-1 nephritis group and the Thy-1 nephritis group by electron microscopy (***Fig. 7***).

### The roles of renal TSP-1 expression in mesangial ECM secretion in the glomeruli of rats with Thy-1 nephritis

The above-mentioned studies have shown that the renal expression of the *TSP-1* gene was required for GMC proliferation in Thy-1 nephritis rats. To further evaluate the roles of the *TSP-1* gene in ECM secretion of Thy-1 nephritis rats, the renal expression levels of fibronectin and collagen IV in the 4 groups were determined on d 7 after the induction of nephritis, and the results revealed that shTSP-1 treatment could remarkably inhibit the expression of fibronectin and collagen IV, while shCTR had no significant influence on it (***Fig. 3***). Meanwhile, electron microscopy revealed that the glomeruli of rats in the shTSP-1 + Thy-1 nephritis group showed less ECM accumulation within the mesangia of rats compared to the shCTR + Thy-1 nephritis group and Thy-1 nephritis group (***Fig. 7***).

## DISCUSSION

Human mesangioproliferative glomerulonephritis is a disease with a high incidence, and the proliferation of GMC in mesangioproliferative glomerulonephritis appears to be important in subsequent ECM deposition[1]–[3]. Rat Thy-1 nephritis is a typical animal model for studying human mesangioproliferative glomerulonephritis[6],[7]. Administration of anti-Thy-1 antibody into rats binding to the surface of GMC can lead to complement system activation, resulting in GMC proliferation and ECM secretion during the process of Thy-1 nephritis[34],[35]. However the mechanisms of GMC proliferation and ECM accumulation in Thy-1 nephritis rats have not been well elucidated.

### The effects of *TSP-1* gene knockdown in renal tissues on urinary protein excretion of rats with Thy-1 nephritis

**Fig. 8:**
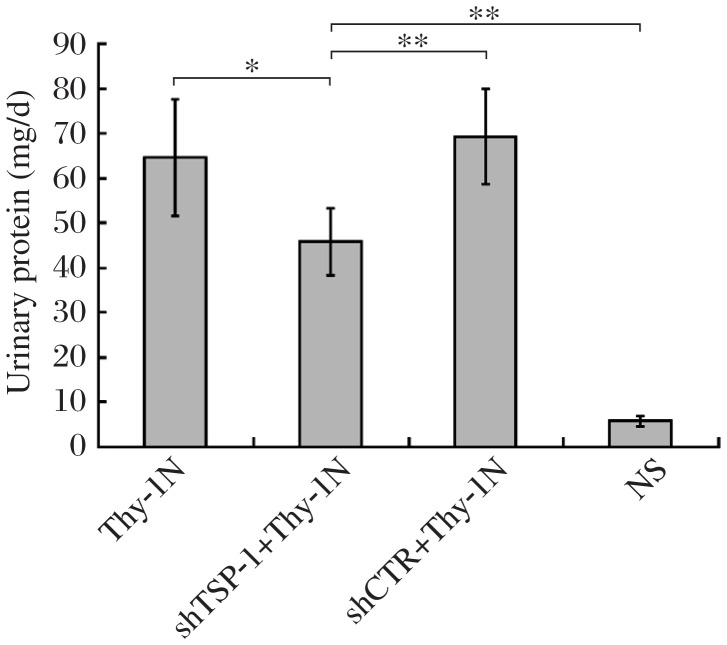
The effect of shTSP-1 on urinary protein secretion (mg/24h) of Thy-1 nephritis rats. The urine samples of rats in different groups were collected on d 7 after the induction of nephritis. The contents of urinary protein of rats were then measured. The data demonstrated that the content of urinary protein from the rats in the shTSP-1 + Thy-1 nephritis group was obviously lower than that in the shCTR + Thy-1 nephritis and Thy-1 nephritis groups on d 7 after the induction of nephritis. Data are represented as mean±SD (*n* = 6 per group). *P < 0.05, **P < 0.01.

The effects of renal *TSP-1* gene silencing on the excretion of urinary protein of Thy-1 nephritis rats were detected, and the results showed that shTSP-1 reduced the content of urinary protein (mg/24 h) of Thy-1 nephritis rats on d 7 after induction of nephritis compared with shCTR treatment (***Fig. 8***), implicating that inhibition of the renal *TSP-1* gene could not only suppress proliferative pathologic changes, but also ameliorates the renal function of Thy-1 nephritis rats.

The deposition of C5b-9, especially sublytic C5b-9 complexes, has been observed in the glomeruli of patients with mesangioproliferative glomerulonephritis and rats with Thy-1 nephritis[4],[29], suggesting that sublytic C5b-9 could be involved in GMC pathological changes. Our previous studies have demonstrated that sublytic C5b-9 attack *in vitro* could cause obvious GMC proliferation and ECM production as well as TSP-1 production. Treatment of GMCs with the GGWSHW peptide (the selective inhibitor of TSP-1) could block the interaction between TSP-1 and TGF-β1 protein and subsequent TGF-β1 activation as well as ECM production[17],[18], indicating that TSP-1 could activate TGF-β1 protein secreted by sublytic C5b-9-stimulated GMC, which in turn promoted ECM synthesis[18]. In addition, our previous studies showed that TSP-1 shRNA could suppress GMC proliferation induced by sublytic C5b-9 *in vitro*.

To reveal the roles of the *TSP-1* gene in the renal proliferative damages of Thy-1 nephritis rats, shTSP-1 expression plasmids were transfected into rat kidneys followed by Thy-1 nephritis induction. Then, we observed the effects of TSP-1 gene knockdown in the renal tissues of rats on GMC proliferation and ECM expansion in Thy-1 nephritis rats. The results displayed that silencing of the *TSP-1* gene could not only abolish GMC proliferation and but also suppress mesangial ECM deposition in Thy-1 nephritis rats, indicating that *TSP-1* gene expression was involved in the proliferative lesions of Thy-1 nephritis rats. On the other hand, the effect of shTSP-1 on the urinary protein secretion of Thy-1 nephritis rats was determined, and the results showed that *TSP-1* gene silencing could markedly reduce urinary protein secretion (mg/24 h) of Thy-1 nephritis rats, which was in agreement with the above-mentioned pathological changes.

Interestingly, the findings in our present study were not completely consistent with that in Daniel's study[27], as it was shown that the transfer of oligonucleotide against TSP-1 could inhibit glomerular ECM accumulation, but had no significant influence on GMC proliferation of Thy-1 nephritis rats. The difference between the two studies might explain that in our studies, and shTSP-1 expression plasmids were used and transferred into the kidneys on d 3 before induction of Thy-1 nephritis, but not on d 2 after Thy-1 nephritis induction. The suppression of renal TSP-1 gene ahead of the nephritis could affect some other factors or signaling transduction pathways, which could regulate GMC proliferation. Further studies need to be done exploring the mechanism through which *TSP-1* regulates GMC proliferation in Thy-1 nephritis rats.

In conclusion, these findings confirm that *TSP-1* gene expression was required for GMC proliferation and ECM secretion as well as urinary protein excretion in rat Thy-1 nephritis, which might provide novel insights into the pathogenesis of human mesangioproliferative glomerulonephritis.
